# Hydrogen‐Rich Saline Combined With Vacuum Sealing Drainage Promotes Wound Healing by Altering Biotin Metabolism

**DOI:** 10.1111/jcmm.70292

**Published:** 2025-01-13

**Authors:** Xinwen Kuang, Zhengyun Liang, Yijun Xia, Mengjie Shan, Yan Hao, Hao Liu, Zhi Wang, Qianjun He, Chao Xia, Cheng Feng, Guojing Chang, Youbin Wang

**Affiliations:** ^1^ Department of Plastic Surgery, Peking Union Medical College Hospital Chinese Academy of Medical Science and Peking Union Medical College Beijing China; ^2^ Department of Dermatology, Shenzhen Center for Chronic Disease Control Shenzhen Institute of Dermatology Shenzhen China; ^3^ Shanghai Key Laboratory of Hydrogen Science & Center of Hydrogen Science, School of Materials Science and Engineering Shanghai Jiao Tong University Shanghai China

**Keywords:** biotin metabolism, hydrogen‐rich saline, metabolomics, oxidative stress, vacuum sealing drainage, wound healing

## Abstract

Impaired wound healing affects the life quality of patients and causes a substantial financial burden. Hydrogen‐rich medium is reported to have antioxidant and anti‐inflammatory effects. However, the role of hydrogen‐rich saline (HRS) in cutaneous wound healing remains largely unexplored, especially by metabolomics. Thus, untargeted metabolomics profiling was analysed to study the effects and mechanism of HRS combined with vacuum sealing drainage (VSD) in a rabbit full‐thickness wound model. Our results indicated that the combination treatment of HRS and VSD could accelerate wound healing. In vitro experiments further confirmed its effects on HaCaT keratinocytes. We found that 45 metabolites were significantly changed between the VSD + HRS group and the VSD + saline‐treated group. Pathway enrichment analysis indicated that biotin metabolism was the potential target pathway. The biochemical interpretation analysis demonstrated that combining HRS and VSD might enhance mitochondrial function, ATP synthesis, and GSH homeostasis by altering biotin metabolism. The detection of representative indicators of oxidative stress supported the critical metabolic pathway analysis as well. In summary, VSD combined with HRS might provide a new strategy to enhance wound healing.

**Abbreviations**
CAT: catalase
FDR: false discovery rate
GSH: glutathione
HE: haematoxylin ANeosin
HRS: hydrogen‐rich saline
IL‐10: interleukin 10
IL‐1β: interleukin 1β
MDA: malondialdehyde
NPWT: negative pressure wound therapy
PCA: principal component analysis
PyroGlu: pyroglutamic acid
qRT‐PCR: quantitative real‐time PCR
ROS: reactive oxygen species
SD: standard deviations
SOD: superoxide dismutase
TCA: tricarboxylic acid
TNF‐α: tumour necrosis factor‐α
Tukey HSD: Tukey honest significant difference
VIP: variable importance in the projection
VSD: vacuum sealing drainage

## 1 Introduction

The skin covers the surface of the human body and serves as the first line of defence against the external environment [1]. Wound healing is a complex, multifactorial process that occurs when skin integrity is compromised [2]. While mild skin injuries can gradually recover completely, numerous factors, such as infection, insufficient angiogenesis, and excessive oxidative stress, can result in delayed wound healing [3, 4], adversely impacting patients' quality of life [5]. Furthermore, chronic wounds pose a significant financial burden worldwide [6], highlighting the need for effective interventions to accelerate wound repair.

Wound healing involves four phases: coagulation and haemostasis, inflammation, proliferation (neo‐angiogenesis, granulation, re‐epithelialisation), and maturation with scar tissue formation. Completing these phases requires high energy since the wound environment is highly hypoxic [7]. Both local factors and the physiological status contribute to wound healing [8, 9], making local metabolic balance essential for effective cutaneous tissue repair.

Biotin, a key cofactor for enzymes involved in glucose, amino acid, and fatty acid metabolism, is essential for energy metabolism and cellular growth [10, 11]. Biotin deficiency can hinder cell proliferation, resulting in reduced collagen synthesis and, immune dysfunction [12]. The importance of biotin has been demonstrated across various skin cell types, including fibroblasts [13, 14]. Additionally, untreated biotin deficiency can lead to cutaneous symptoms such as seborrhoeic eczema, dermatitis, and alopecia [15, 16]. Given the energy demands of wound healing, biotin metabolism and recycling may play a crucial role in supporting the repair process.

Hydrogen is a gas with antiapoptotic, anti‐inflammatory, and antioxidant properties. Our research team previously discovered the protective effects of inhaled hydrogen on skin injury [17] and the wound‐healing effects of molecular hydrogen [18]. However, the storage and transportation of hydrogen is not conducive to its clinical usage [19]. Alternative administration routes, such as oral intake of hydrogen‐rich water and injection of hydrogen‐rich saline (HRS), offer safe and effective methods for delivering molecular hydrogen. Previous studies have confirmed the antioxidant effects of the hydrogen‐rich medium in various diseases, such as inflammatory bowel disease and sepsis [20, 21, 22, 23, 24, 25, 26, 27, 28, 29, 30]. Recent studies in the field of skin defects have further indicated the therapeutic potential of HRS [31].

Vacuum sealing drainage (VSD) is an effective technique that uses vacuum dressings to accelerate wound healing [32]. However, few studies have investigated the potential of HRS to accelerate wound healing or its capacity to maintain redox and metabolic balance at the wound site, especially in combination with VSD. While metabolomics approaches have been utilised to characterise the metabolic profile of wounds [33, 34], there is a lack of convincing studies on the wound‐healing effect of HRS combined with VSD using metabolomics. Therefore, the purpose of this study was to assess the effect of combination treatment of HRS and VSD on wound healing. Untargeted metabolomics was employed to explore the metabolic changes caused by HRS combined with VSD and elucidate the underlying mechanisms.

## 2 Materials and Methods

### 2.1 Ethics Statement

The animal study protocol was approved by the Ethics Committee of the Chinese Academy of Medical Sciences and Peking Union Medical College Hospital (ethics number: XHDW‐2023‐063).

### 2.2 Wound Creation and Grouping

Male New Zealand white rabbits aged 80 days were purchased from the Fangyuanyuan Breeding Farm of Beijing. All animals were single‐housed at room temperature for 5 days prior to surgery. One day before surgery, they remained fasted (with free access to water) for 12 h. Before surgery, the dorsum of the rabbits was shaved with Veet. The rabbits were placed in a prone position after anaesthesia with Zoletil 50 (50 mg/mL, 3 mg/kg). Two surgical sites were marked using a circular standard template 2.0 cm in diameter at both sides of the back of the rabbits. The subcutaneous superficial layer of fascia was bluntly separated using sterile scissors following a surgical incision with a sterile surgical blade. As a final step, the 2 cm‐diameter circular skin was cut off completely to construct the full‐thickness wound model. Dressings made of sterile gauze were applied to the wounds. All procedures were performed by the same surgeon. The animals were randomised into five groups of 6 rabbits each: Control, Saline, HRS, VSD + Saline, and VSD + HRS.

### 2.3 Preparation of HRS

HRS was produced in 280 mL using a hydrogen generator (Nanobarber) and dissolved in physiological saline under 0.4 MPa pressure at room temperature. To ensure the concentration of hydrogen in the HRS, all reagents were used right after preparation.

### 2.4 Wound Therapy

Wounds in the VSD + Saline and VSD + HRS groups were treated by the negative pressure wound therapy (NPWT) device (Daewoong Pharm) with a negative pressure of 125 mmHg (Figure S1A). Since the first day after modelling, the rabbits in VSD + Saline and the VSD + HRS groups underwent a 2‐h treatment each day for 7 days [35]. For HRS and VSD + HRS groups, each wound received HRS irrigation three times daily for 7 days (each time for 3 min). The wounds in the saline group received saline irrigation for 3 min three times daily for 7 days. The control group received no additional treatment. A ratio of the remaining wound area to the original wound area was used to calculate the percentage of wound healing [36].

### 2.5 Haematoxylin–Eosin Stainings

For histochemical analyses, wound tissue specimens from New Zealand rabbits were fixed in formalin and routinely embedded in paraffin. 5 mm thick paraffin sections were processed for haematoxylin–eosin (HE) staining according to the standard protocol. HE staining was imaged with a Pannoramic MIDI system (3DHISTECH).

### 2.6 RNA Isolation and Quantitative Real‐Time (qRT)‐PCR

IL‐1β, TNF‐α, and IL‐10 levels were assessed to determine the local inflammation in wound tissues. PCR primers are listed in Table S1. The extracted RNA was subsequently quantified using a Nanodrop ND‐2000 spectrophotometer (Thermo Fischer Scientific). Following the manufacturer's protocol, first‐strand cDNA was synthesised from 500 ng RNA using HiScript III RT SuperMix (R323‐01, Vazyme, Nanjing, China) for qPCR. qRT‐PCR was conducted using SYBR green reagents (SYBRTM Green master mix, Q711‐02‐AA, Vazyme, Nanjing, China). A comparative Ct analysis was used, and β‐actin was used as a housekeeping reference gene. Each sample was analysed for gene expression in at least triplicate.

### 2.7 Cell Culture and CCK‐8 Assay

The human keratinocyte cell line HaCaT [37] was grown in a 37°C humidified chamber with 5% CO_2_ in DMEM medium (Gibco) with 10% foetal bovine serum (Gibco) and 1% mixture of penicillin–streptomycin (Gibco). The cells were divided into four groups: saline, HRS, VSD + saline and VSD + HRS. For the HRS and VSD + HRS groups, 10% HRS was added; for the saline and VSD + saline groups, an equivalent volume of normal saline was added. To estimate the effects of VSD, we assembled the bioreactor modifying from the previous report [38] and a natural inhibitor of IL‐1β, IL‐1RA (10 ng/mL, Novoprotein), was added [39, 40]. Each group was grown for 24 h at 37°C with 5% CO_2_. 10 μL CCK‐8 (Solarbio) reagent was then used, and the plate was cultured for an additional 2 h after that. The absorbance was measured at 450 nm to test the cell viability using the Synergy H1 plate reader (BioTek). Cell viability = (absorbance of experimental wells‐absorbance of blank wells)/(absorbance of control wells‐absorbance of blank wells) * 100%. The experiment was repeated three times.

### 2.8 Samples Collection and Metabolites Extraction

Each wound tissue specimen (100 mg) was ground with liquid nitrogen and resuspended in prechilled 80% methanol by well vortex. In this study, wound tissue specimens were collected on day 10 after the modelling process for metabolomics analysis. The samples were incubated on ice for 5 min and centrifuged for 20 min at 15,000 *g*, 4°C. Then we diluted some supernatant with a final concentration containing 53% methanol in LC–MS grade water. After being transferred to a fresh Eppendorf tube, the specimens were centrifuged at 15,000 *g*, 4°C for 20 min. Finally, the supernatant was injected into the LC–MS/MS system analysis [41].

### 2.9 Non‐Targeted Metabolomics Analysis

UHPLC–MS/MS analyses were conducted by a Vanquish UHPLC system (Thermo Fisher) coupled with an Orbitrap Q ExactiveTM HF mass spectrometer or Orbitrap Q ExactiveTM HF‐X mass spectrometer (Thermo Fisher). Using a 12‐min linear gradient at a flow rate of 0.2 mL/min, each wound tissue specimen was injected into a Hypersil Gold column (100 × 2.1 mm, 1.9 μm). The eluents for the positive polarity mode were eluent A (0.1% FA in water) and eluent B (Methanol). The eluents for the negative polarity mode were eluent A (5 mM ammonium acetate, pH 9.0) and eluent B (Methanol). The solvent gradient was set as follows: 2% B, 1.5 min; 2%–85% B, 3 min; 85%–100% B, 10 min; 100%–2% B, 10.1 min; 2% B, 12 min. In the positive/negative polarity mode, the Q ExactiveTM HF mass spectrometer was operated at 3.5KV, with a 320°C capillary temperature, 35 PSI sheath gas flow rate, 10 L/min aux gas flow rate, 60 RF level of S‐lens and 350°C aux gas heater temperature.

### 2.10 Multivariate Data Processing and Data Analysis

To perform peak alignment, peak picking, and quantitation for each metabolite, the raw data files generated by UHPLC–MS/MS were processed using the Compound Discoverer 3.3 (CD3.3, Thermo Fisher). We set the following main parameters: peak area was corrected with the first QC; actual mass tolerance, 5 ppm; signal intensity tolerance, 30%; and minimum intensity, et al. Then, peak intensities were normalised to the total spectral intensity. The normalised data was utilised to speculate on the molecular formula based on additive ions, molecular ion peaks, and fragment ions. Then the peaks were matched with the mzCloud (https://www.mzcloud.org/), mzVault, and MassList databases to obtain accurate qualitative and relative quantitative results.

When data were not normally distributed, they were standardised according to the formula: sample raw quantitation value/(the sum of sample metabolite quantitation value/the sum of QC1 sample metabolite quantitation value) to obtain relative peak areas. And we removed the compounds whose CVs of relative peak areas in QC samples were greater than 30%. Finally, the metabolites' identification and relative quantification results were obtained.

### 2.11 Differential Metabolites Identification and Pathway Analysis

Metabolic and reconstruction of pathway metabolites of interest were extracted from OPLS‐DA score plots. The ion spectra were matched with the structural message of metabolites acquired from biochemical databases, such as the KEGG database (https://www.genome.jp/kegg/pathway.html) and HMDB database (https://hmdb.ca/metabolites). The reconstruction pathway analysis was conducted based on the above database sources.

### 2.12 Determination of Superoxide Dismutase, GSH, Catalase, and Malondialdehyde

Superoxide dismutase (SOD), GSH, catalase (CAT), and malondialdehyde (MDA) detection kits were purchased from Solarbio. The content of SOD, GSH, CAT, and MDA was measured according to the manufacturer's instructions. The absorbance was determined using a Synergy H1 plate reader (BioTek).

### 2.13 Statistical Analysis

Mean values are expressed as mean ± SD unless otherwise stated. Two‐sided *p* values at or below 0.05 were considered statistically significant. Statistical analyses were carried out in R (version 3.6.3) and CentOS (CentOS release 6.6); data visualisation was presented using the Wekemo Bioincloud (https://www.bioincloud.tech).

## 3 Results

### 3.1 The Combination Therapy of HRS and VSD Accelerated Wound Healing in a Rabbit Model

**FIGURE 1:**
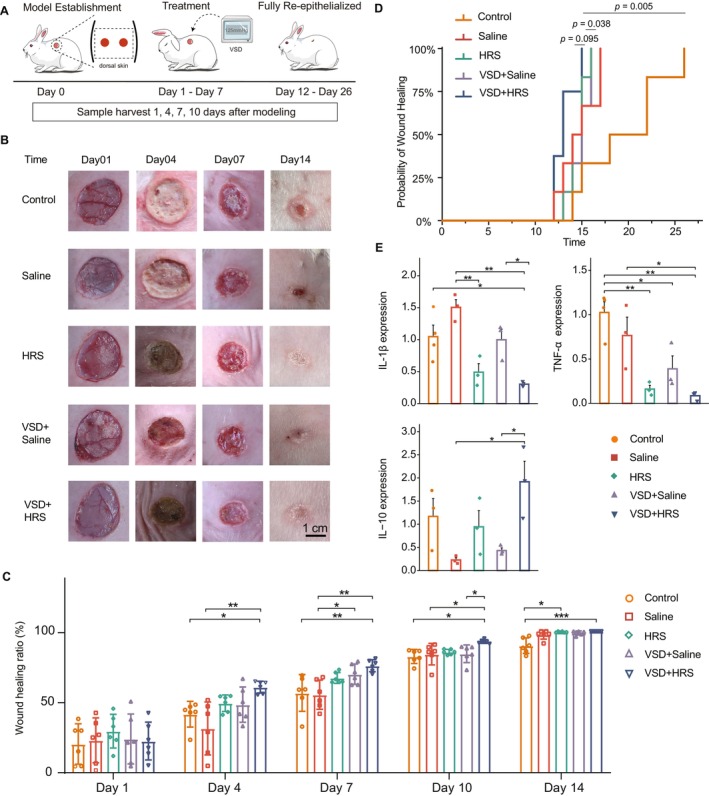
(A) The schedule of a rabbit full‐thickness wound model establishment, the treatment, and the sample harvest. (B) Representative wound pictures of rabbits' dorsal from each group were taken on post‐injury days 1, 4, 7, and 14. Scale bar, 1 cm. (C) Wound healing rate at different times from each group. Data are shown as means ± SD (*n* = 6–8). Variables in the five groups were compared by the one‐way ANOVA test, Welch's ANOVA, and the Kruskal–Wallis rank‐sum test. The Games‐Howell test (a post hoc test) and Dunn's test were used for multiple comparisons when appropriate. (D) The time to complete wound closure in different groups was measured and compared. The Kaplan–Meier survival curve was generated, and the Chi‐square test analysed statistical differences. (E) Expression of cytokines, TNF‐α, IL‐1β, and IL‐10. *n* = 3–4. Variables in the five groups were compared using the one‐way ANOVA test. The Tukey honest significant difference (Tukey HSD) test was used for multiplecomparisons. ***, **, and * represent significance at the significance level of 1%, 5%, and 10% respectively.

The design of the in vivo study is outlined in Figure 1A. After establishing the full‐thickness skin defects model, the rabbits were divided into 5 groups: a control group with no treatment, a saline rinse group, an HRS rinse group, a VSD + saline group, and a VSD + HRS group. To investigate the effect of HRS on wound healing, we recorded the wound healing rates on the 1st, 4th, 7th, 10th, and 14th days after different interventions. In the HRS and VSD + HRS groups, each wound was rinsed three times daily for 3 min with HRS to allow it to take full effect. The Saline and VSD + Saline groups were washed with saline at the same frequency. Macroscopic observation of the wound tissue between different groups showed no apparent change (*p* > 0.05) in the wound morphology at day 1. On the 4th, 7th and 10th days, the wound healing rate in the VSD + HRS group was significantly higher (*p* < 0.05) than in the control and saline groups (Figure 1C).

We also observed that wound closure had accelerated significantly (*p* = 0.0466) in the VSD + Saline group compared to that in the Saline group on the seventh day. These results indicated that combining VSD and HRS might enhance wound healing. Of note, the wound healing rate of the VSD + HRS group was statistically higher (*p* = 0.0484) than that of the VSD + Saline group at day 10. Besides, throughout the experimental timeline, the wound healing rate was better with the combination therapy of VSD + HRS (with an average healing rate of 60.555% at day 4, 75.711% at day 7, and 99.997% at day 14) than with the combination therapy of VSD + saline (with an average healing rate of 48.207% at day 4, 69.724% at day 7, and 98.848% at day 14). It suggested that HRS enhances the effect of VSD.

We also measured the time to complete closure of each wound. Similarly, the resulting time‐to‐closure functions for the HRS and VSD + HRS‐treated wounds were significantly distinct (*p* = 0.022, *p* = 0.005, respectively) from the control group (Figure 1D). These results further illustrated the therapeutic effect of the HRS. Compared to the VSD + HRS group, the VSD + Saline and HRS groups prolonged wound closure time (*p* = 0.038, *p* = 0.095, respectively). This result further proved that VSD and HRS can promote each other's effect in terms of wound healing.

Cytokines, including TNF‐α, IL‐1β, and IL‐10, play a role in tissue repair [42]. Hence, the levels of cytokines were determined to assess the anti‐inflammatory effects of the VSD + HRS treatment (Figure 1E). A qRT‐PCR analysis suggested that the mRNA expression levels of pro‐inflammatory cytokines, TNF‐α and IL‐1β, were significantly downregulated in the VSD + HRS group compared to the Control and Saline groups (Figure 1E). As expected, combination therapy with VSD and HRS significantly decreased the IL‐1β levels of the wound compared to the VSD + saline treatment. These results indicate that VSD and HRS combination therapy markedly promotes wound healing, partly due to their anti‐inflammatory effects. We also observed that IL‐10 levels were significantly increased in the VSD + HRS group compared to the saline and VSD + saline‐treated groups (Figure 1E). Considering that IL‐10 can modulate the expression of pro‐inflammatory cytokines that reduce tissue damage during the inflammation resolution phase [43], these results suggest that VSD and HRS combination therapy may regulate local inflammation to accelerate wound repair.

### 3.2 The Combination Therapy of HRS and VSD Promoted Re‐Epithelialisation

**FIGURE 2:**
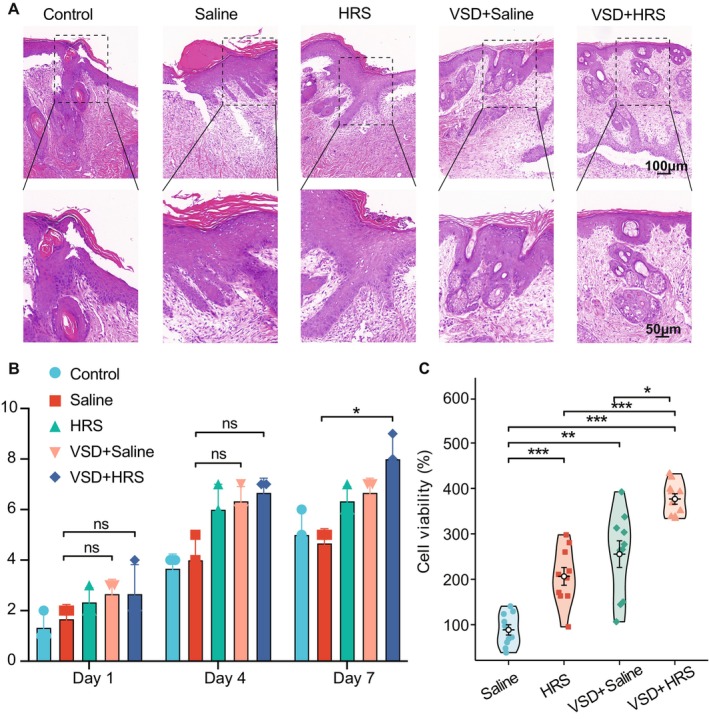
(A) HE staining of wound section on day 7. The scale bar of the upper panel is 100 μm; the lower, 50 μm. (B) Histopathological scores were calculated by HE staining on days 1, 4, and 7. (*n* = 3, data = mean ± SD.) Variables in the five groups were compared using the Kruskal–Wallis rank‐sum test. Dunn's test was used for multiple comparisons. (C) Cell viability on HaCaT cells with different interventions was evaluated by CCK‐8 assay. (*n* = 10, data = mean ± SEM.) Variables in the four groups were compared using the Welch one‐way ANOVA test. A Games‐Howell post hoc analysis was applied to determine multiple comparisons. ***, **, and * represent significance at the significance level of 1%, 5%, and 10% respectively.

The wound healing quality was further evaluated through HE staining. Figure 2A showed pathological changes in the wounds on day 7 post‐modelling. The wound healing quality in the VSD + Saline group and VSD + HRS group was better than in the Control and Saline groups (Figure 2A), with more capillaries, thicker granulation tissues, and obvious proliferation. Based on the previous research [44], histopathological scores were calculated (Figure 2B). The evaluation includes epidermal structure, dermal‐epidermal junction, collagen bundles, epidermal regeneration, and leukocyte infiltration. And the presence of normal structures assigned the highest scores. The semiquantitative results were consistent with the observed wound‐healing trends. Notably, the scores in the VSD + HRS group were significantly higher than in the saline group (*p* = 0.0379). Additionally, there was a trend that VSD + HRS‐treated samples were better than those treated with VSD + saline, demonstrating that HRS strengthens the effects of VSD.

### 3.3 Effects of the Combination Therapy of HRS and VSD on HaCaT Cell Function

According to the findings, the combination therapy of HRS and VSD would accelerate wound healing in animal models. Preliminary findings suggested that the combination treatment may regulate local inflammation and promote re‐epithelialisation. Hence, in vitro cell experiments were conducted to verify the effect of the combination treatment on cell biological functions. Based on the treatments, HaCaT cells were grouped into Saline, HRS, VSD + Saline, and VSD + HRS, respectively. The CCK‐8 assay results illustrated that cell growth was significantly increased by VSD and HRS combination therapy when compared to the other three groups (Figure 2C, *p* < 0.05). Besides, treatment of HRS and VSD + Saline could significantly promote the rate of cell viability at 24 h (*p* = 0.0006, *p* = 0.0010, respectively) compared to saline treatment.

### 3.4 Quality Assessment and QC of Wound Samples

Next, we employed a non‐targeted metabolomics technique to characterise the tissue metabolome. Before analysis, we conducted data preprocessing, which included quality control and normalisation. PC1 values were analysed to evaluate the controllability of the sample preparation and measurement process. The result revealed that most points would fluctuate up and down around the axis within two standard deviations above the mean (2SDs) (Figure S1B). Using the QC‐RFSC algorithm, the characteristic signal peaks of each sample (each metabolite) were corrected, and the correcting effect of each metabolite was measured. To ensure the accuracy of the later analysis of metabolite importance, we then normalised the metabolite mean and SD to the same level (Figure S1C).

### 3.5 Overall Metabolomics Analysis of Wound Samples

**FIGURE 3:**
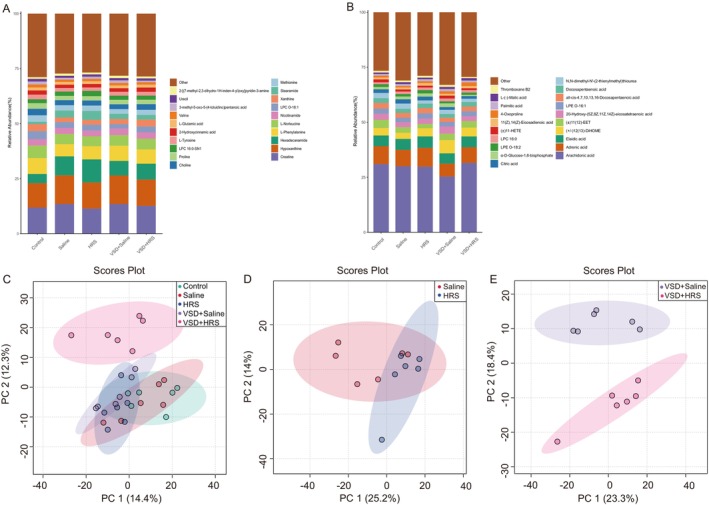
The column graphs show the top 20 content of metabolites in positive (A) and negative ion mode (B). The unsupervised multivariate principal component analysis (PCA) of the studied metabolites in the 5 groups (C). The plots represent the first principal component (PC1) against the second principal component (PC2). The unsupervised data analysis between the Saline group and HRS group (D) and between the VSD + Saline group and the VSD + HRS group (E).

A total of 912 and 669 attributes were collected in the ESI^+^ (positive) and ESI^−^ (negative) modes. Figure 3A,B show the top 20 content percentages of metabolites in positive and negative ion modes, respectively. Since positive ion mode detected more metabolites, subsequent analyses used positive ion mode data. The principal component analysis (PCA) was utilised (Figure 3C) for multivariate analysis. Of note, PCA analysis illustrated significant separation of the VSD + HRS group from the other four groups. To investigate the effects of HRS, PCA was used to perform unsupervised data analysis between the Saline and HRS groups (Figure 3D). And the same analysis was performed on the VSD + Saline group and VSD + HRS group (Figure 3E).

**FIGURE 4:**
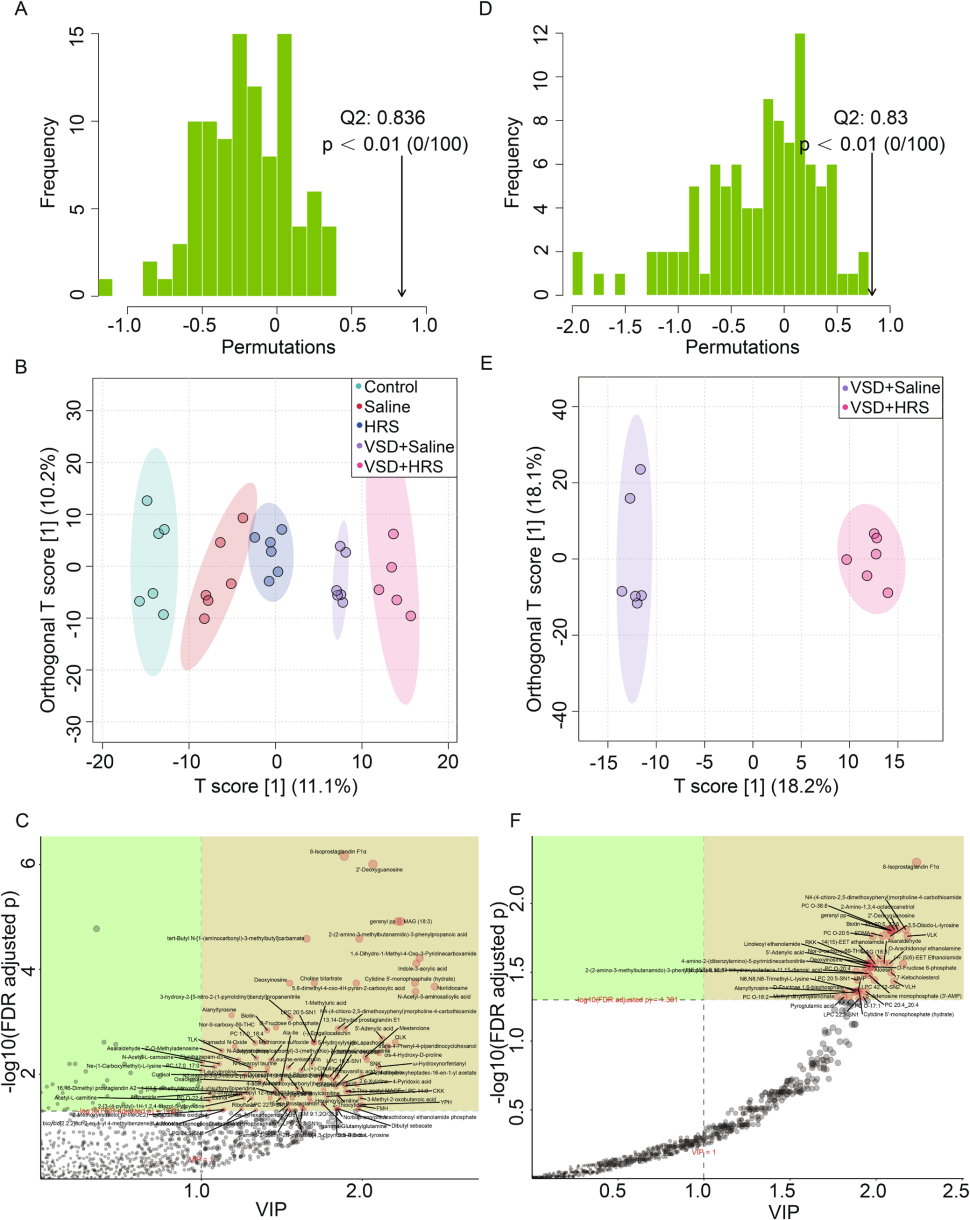
The Permutation test implicated the validity of the OPLS‐DA model among all groups (A) and between the VSD + Saline group and VSD + HRS group (B). OPLS‐DA score plots among all groups (C) and between the VSD + Saline and VSD + HRS groups (D). Variable importance in the projection (VIP) values of OPLS‐DA analysis among all groups (E) and between the VSD + Saline group and the VSD + HRS group (F). The points represent the metabolites, and the abscissa and ordinate represent the Value Importance in Projection (VIP) values and −log_10_ (FDR‐adjusted *p*‐value) of the quantitative difference of metabolites.

Then, supervised analysis OPLS‐DA (Figure 4) was utilised to identify additional metabolites that were not detected by PCA. The permutation testing of the OPLS‐DA model (Figure 4A,D) indicated that the models were reliable and not overfitted. The OPLSDA scores plot (Q2 = 0.830, *p* < 0.01, Figure 4E) demonstrates a clear separation between the VSD + HRS and VSD + Saline groups. In detail, variable importance in the projection (VIP) values of OPLS‐DA analysis was shown in Figure 4C,F.

According to PCA and OPLS‐DA score plots, there were clear differences in metabolic profiles between the VSD + HRS and VSD + Saline groups at baseline. Taken together, the analysis indicated that the potential mechanism of the VSD + HRS treatment to accelerate wound healing may be related to changes in metabolites.

### 3.6 Detection and Identification of Differential Metabolites

**FIGURE 5:**
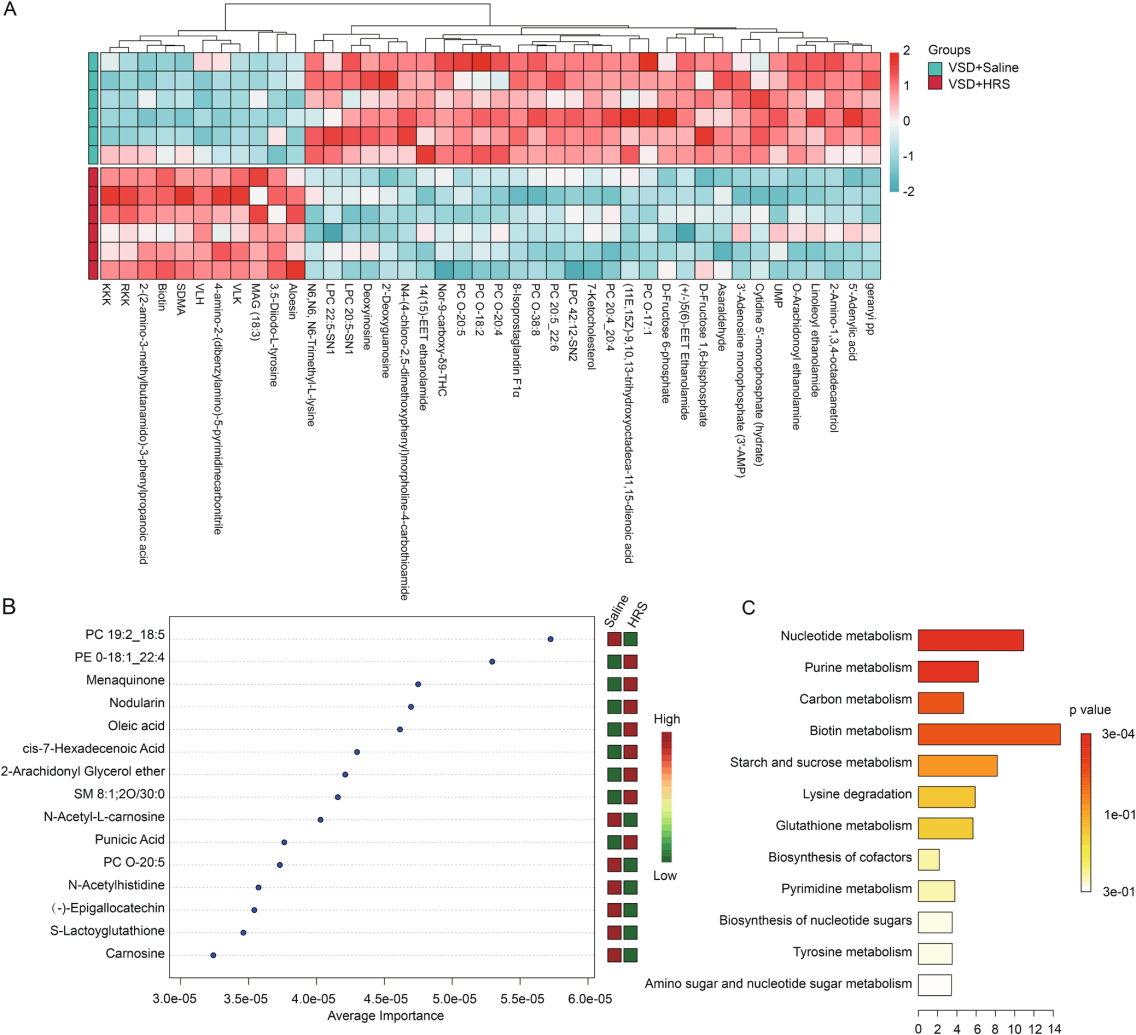
(A) Heat maps of differential metabolites from the cutaneous specimens. Rows: Samples; Columns: Metabolites. Red means the metabolites is expressed at a higher level, and blue means the metabolites is expressed at a lower level. (B) The 15 most important metabolites in the SVM model between HRS and Saline groups. The importance of metabolites was assessed by the degree of average importance (bottom abscissa). The heat map of the 15 most important metabolites was displayed on the right side. Red: Higher expression levels; green: Lower expression levels. (C) KEGG enrichment analysis between the VSD + Saline group and the VSD + HRS group. The horizontal coordinates are fold changes. It refers to the ratio of the differential metabolite numbers in the corresponding pathway to the total identified metabolite numbers in this pathway. The higher the ratio value, the more differential metabolites are enriched in this pathway. The darker the colour, the smaller the *p*‐value.

Then we focused on the comparison results between the VSD + HRS and VSD + Saline groups. To identify differential metabolites significantly associated with VSD and HRS combination therapy, the criteria were selected below: [1] VIP score > 1.0 from the OPLS‐DA model; [2] *p*‐value < 0.05 and false discovery rate (FDR) < 0.05. Then 45 metabolites (Down: 32, Up: 13) were shown in Table 1. To characterise the metabolic profile, we drew a heat map based on the intensity levels of 45 markers (Figure 5A). In particular, there were 10 metabolites linked to known metabolic pathways, including 2′‐Deoxyguanosine, Biotin, 3,5‐Diiodo‐L‐tyrosine, Pyroglutamic acid (PyroGlu), D‐Fructose 6‐phosphate, Deoxyinosine, 5′‐Adenylic acid, UMP, N6,N6,N6‐Trimethyl‐L‐lysine, and D‐Fructose 1,6‐bisphosphate.

**TABLE 1:** Identified potential biomarkers. Up indicates increase; Down indicates decrease.

Formula	Biomarker	VIP	*p*	FDR	VSD + HRS vs. VSD + Saline
C20 H36 O5	8‐Isoprostaglandin F1α	2.235	0.000	0.005	Down
C17 H34 N4 O4	VLK	2.183	0.000	0.017	Up
C13 H17 Cl N2 O3 S	N4‐(4‐chloro‐2,5‐dimethoxyphenyl)morpholine‐4‐carbothioamide	2.171	0.000	0.015	Down
C22 H37 N O3	(+/−)5(6)‐EET Ethanolamide	2.156	0.001	0.027	Down
C10 H13 N5 O4	2′‐Deoxyguanosine	2.125	0.000	0.015	Down
C18 H39 N O3	2‐Amino‐1,3,4‐octadecanetriol	2.105	0.000	0.015	Down
C17 H29 N5 O4	VLH	2.104	0.001	0.037	Up
C46 H78 N O7 P	PC O‐38:8	2.089	0.000	0.016	Down
C27 H44 O2	7‐Ketocholesterol	2.088	0.001	0.032	Down
C10 H16 N2 O3 S	Biotin	2.086	0.000	0.016	Up
C9 H9 I2 N O3	3,5‐Diiodo‐L‐tyrosine	2.082	0.000	0.017	Up
C50 H78 N O8 P	PC 20:5_22:6	2.082	0.000	0.015	Down
C22 H37 N O2	O‐Arachidonoyl ethanolamine	2.078	0.001	0.027	Down
C10 H20 O7 P2	geranyl pp	2.063	0.000	0.015	Down
C10 H12 O4	Asaraldehyde	2.046	0.001	0.027	Down
C8 H18 N4 O2	SDMA	2.037	0.000	0.017	Up
C21 H36 O4	MAG (18:3)	2.026	0.000	0.025	Up
C21 H28 O4	Nor‐9‐carboxy‐δ9‐THC	1.993	0.001	0.027	Down
C50 H78 N O7 P	LPC 42:12‐SN2	1.990	0.001	0.036	Down
C28 H48 N O7 P	PC O‐20:5	1.980	0.000	0.017	Down
C6 H13 O9 P	D‐Fructose 6‐phosphate	1.980	0.001	0.029	Down
C19 H22 O9	Aloesin	1.970	0.001	0.029	Up
C22 H37 N O3	14(15)‐EET ethanolamide	1.970	0.000	0.025	Down
C18 H38 N8 O4	RKK	1.968	0.001	0.027	Up
C10 H12 N4 O4	Deoxyinosine	1.965	0.001	0.029	Down
C10 H14 N5 O7 P	3′‐Adenosine monophosphate (3′‐AMP)	1.962	0.001	0.033	Down
C14 H20 N2 O3	2‐(2‐amino‐3‐methylbutanamido)‐3‐phenylpropanoic acid	1.953	0.001	0.032	Up
C20 H37 N O2	Linoleoyl ethanolamide	1.952	0.001	0.027	Down
C10 H14 N5 O7 P	5′‐Adenylic acid	1.946	0.001	0.027	Down
C48 H80 N O8 P	PC 20:4_20:4	1.941	0.002	0.044	Down
C19 H17 N5	4‐amino‐2‐(dibenzylamino)‐5‐pyrimidinecarbonitrile	1.940	0.001	0.029	Up
C25 H50 N O7 P	PC O‐17:1	1.926	0.002	0.048	Down
C18 H32 O5	(11E,15Z)‐9,10,13‐trihydroxyoctadeca‐11,15‐dienoic acid	1.925	0.001	0.032	Down
C9 H14 N3 O8 P	Cytidine 5′‐monophosphate (hydrate)	1.901	0.002	0.043	Down
C30 H52 N O7 P	LPC 22:5‐SN1	1.900	0.002	0.049	Down
C9 H13 N2 O9 P	UMP	1.896	0.002	0.043	Down
C9 H20 N2 O2	N6,N6,N6‐Trimethyl‐L‐lysine	1.893	0.002	0.043	Down
C28 H48 N O7 P	LPC 20:5‐SN1	1.891	0.001	0.036	Down
C28 H50 N O7 P	PC O‐20:4	1.883	0.001	0.031	Down
C6 H14 O12 P2	D‐Fructose 1,6‐bisphosphate	1.879	0.002	0.043	Down
C26 H50 N O7 P	PC O‐18:2	1.874	0.002	0.044	Down
C18 H38 N6 O4	KKK	1.844	0.002	0.048	Up
C12 H16 N2 O4	Alanyltrosine	1.818	0.002	0.043	Up
C5 H7 N O3	Pyroglutamic acid	1.813	0.002	0.048	Up
C13 H22 O3	Methyl dihydrojasmonate	1.773	0.002	0.048	Down

### 3.7 KEGG Pathway Enrichment Analysis

To identify key metabolic pathways involved in the VSD + HRS treatment, a KEGG enrichment analysis was conducted comparing the VSD + Saline and VSD + HRS groups. Fisher's exact method was used to calculate the significance of the enriched pathways. Pathway enrichment analysis identified all matched pathways based on *p*‐values and fold‐changes from pathway topology analysis. Generally, smaller *p*‐values indicate more significant enrichment. Figure 5C suggested that metabolic pathways involving Nucleotide metabolism, Purine metabolism, Carbon metabolism, Biotin metabolism, Starch and sucrose metabolism, Lysine degradation, Glutathione metabolism, Biosynthesis of cofactors, Pyrimidine metabolism, Biosynthesis of nucleotide sugars, Tyrosine metabolism, Amino sugar, and nucleotide sugar metabolism were highlighted as potential therapeutic targets of HRS. Among these, the Biotin metabolism exhibited the highest fold enrichment. Therefore, the Biotin metabolism pathway was identified as the potential target pathway for VSD and HRS combination therapy.

### 3.8 The Combination Therapy of HRS and VSD Reduced Oxidative Damage in Rabbits

**FIGURE 6:**
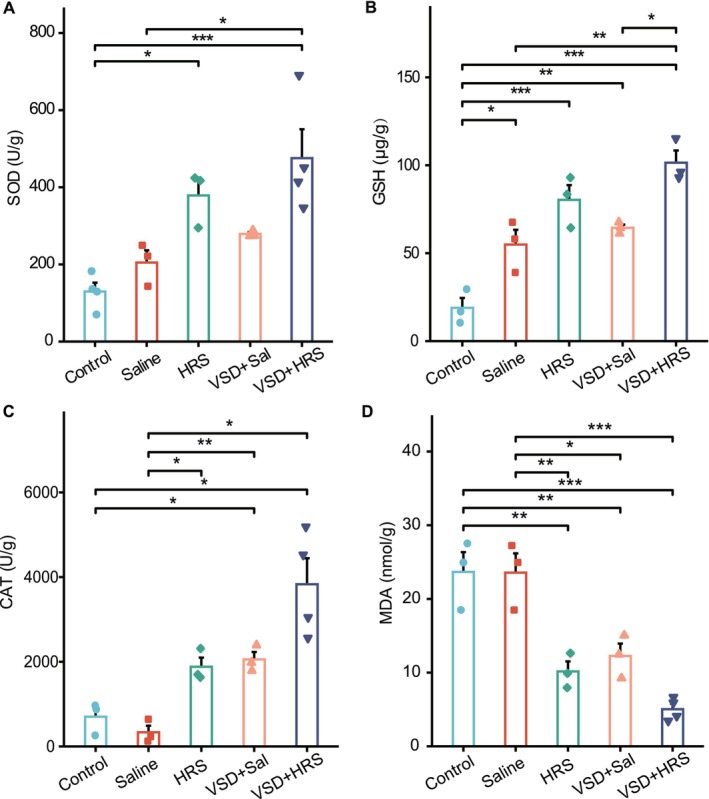
Levels of SOD (A), GSH (B), CAT (C), and MDA (D) content in the wound tissues of different groups. (*n* = 3–4, data = mean ± SEM.) Variables in the five groups were compared using the one‐way ANOVA test and the Welch one‐way ANOVA test. The Games‐Howell test and Tukey HSD test were used for multiple comparisons when appropriate. ***, **, and * represent significance at the significance level of 1%, 5%, and 10% respectively.

Biotin metabolism is known to play a role in regulating cellular oxidative stress [45, 46]. To validate the pathway analysis, representative oxidative damage markers, including SOD, CAT, GSH, and MDA [47] in the wounds, were measured. Figure 6 illustrated that the levels of SOD, GSH, and CAT were significantly higher in the VSD + HRS group compared to the other groups (*p* < 0.05). Additionally, the MDA levels were markedly decreased in the VSD + HRS, VSD + Saline, and HRS groups (*p* < 0.05), while remaining similar to control levels in the Saline group (Figure 6D). These findings indicated that VSD + HRS treatment may reduce oxidative stress by enhancing antioxidant levels and lowering free radicals. Overall, our results imply that the combination therapy of HRS and VSD promotes wound healing by modulating Biotin metabolism and reducing oxidative stress.

## 4 Discussion

In this study, we investigated the effects of the combination treatment of VSD and HRS on accelerating wound healing in a full‐thickness wound healing model in rabbits. In terms of wound healing rate and closure time, wounds in the VSD + HRS group showed better healing compared to the VSD + Saline group. The pathological evaluation through HE staining further confirmed the enhanced therapeutic effects of HRS when combined with VSD. In vitro studies on HaCaT cells further confirmed the findings.

Then UHPLC–MS/MS was used to identify differential metabolites in multiple metabolic pathways. Combined with previous studies, the differential metabolites connected with Biotin metabolism revealed the effect of HRS combined with VSD. Biotin serves as an essential cofactor that participates in amino acid metabolism, fatty acid biosynthesis, and gluconeogenesis [48]. It plays a role in the tricarboxylic acid (TCA) cycle [49] and nutritional biotin deficiency could cause ATP depletion [50, 51]. Optimal wound healing response is an energy‐expensive process that requires cellular energy provided by mitochondrial oxidative metabolism in the form of ATP [52]. Further, as many studies have illustrated, mitochondrial dysfunction, such as accumulated mitochondrial reactive oxygen species (ROS), is closely related to immune cell recruitment, tissue remodelling, and neoangiogenesis during tissue repair [53, 54]. Hence, we speculated that the biotin levels might decrease after the increased ATP consumption during the wound healing process. Consistently, a recent metabolomic analysis revealed the biotin level was diminished in critically ill trauma patients [55]. In addition, biotin is vital in redox balance and could increase the GSH and SOD levels [56]. PyroGlu is one of the main intermediates in the gamma‐glutamyl cycle [57] and the increase in PyroGlu levels leads to an increase in GSH levels. In this study, PyroGlu was considerably increased in the VSD + HRS‐treated group compared to the VSD + Saline group. Hence, we speculated that the HRS + VSD treatment may improve GSH homeostasis by the alternation of the biotin metabolism. In general, our study indicated that VSD + HRS might enhance the level of biotin and then increase the generation of ATP and GSH by modulating energy homeostasis. Further, to verify the Biotin‐related changes, we determined the indicators of oxidative stress. Consistently, the findings detected that the levels of SOD, GSH, and CAT had significantly climbed in the VSD + HRS group.

In the wound‐healing process, the production of ROS is necessary to defend against bacterial pathogens [58]. However, exposure to excessive ROS leads to extensive oxidative stress and hinders wound healing [59]. Previous studies demonstrated that hydrogen‐rich fluids exert a potential effect against oxidative stress in various diseases [25, 26, 60]. However, there currently needs to be convincing literature to connect HRS to wound repair, especially using untargeted metabolomics. Of note is that the use of HRS combined with VSD in wound healing has yet to be investigated. Our research fills the gap in the study of HRS combined with VSD on wound healing, and it proposes some possible mechanisms. Using metabolomics, we explored the effects of the combination therapy of HRS and VSD and possible underlying functional mechanisms. Our results may provide a promising treatment for better wound healing. However, in‐depth functional verification research of medical and cell samples is necessary for future studies.

**FIGURE 7:**
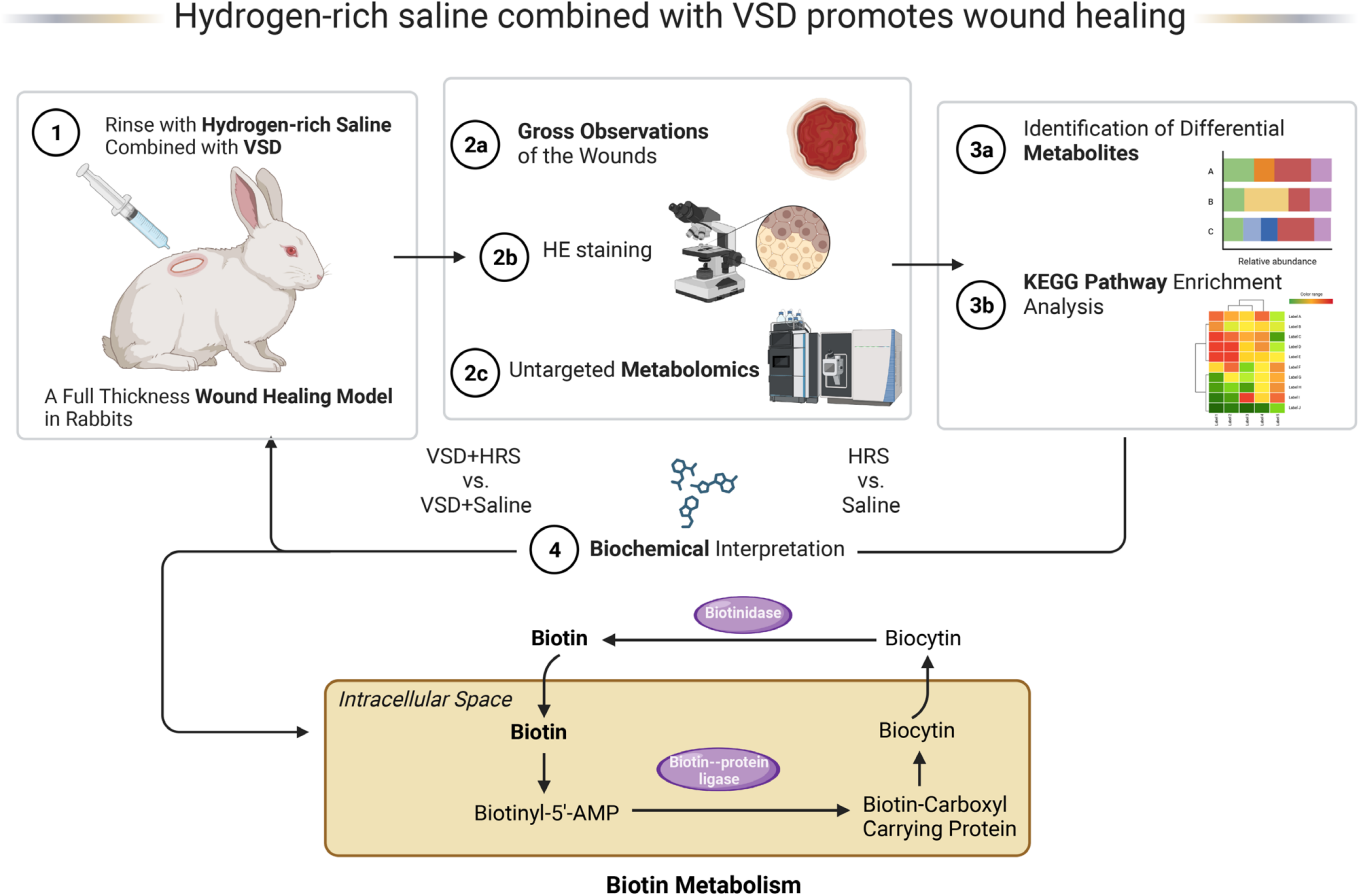
Graphical abstract of the therapeutic effects and the metabolic mechanism of HRS combined with VSD in wound healing (created with BioRender.com; Agreement number: IH267FWL7V).

In general, our study confirmed that VSD combined with HRS can accelerate wound healing, and the combination therapy may function by altering the Biotin metabolism in the wound site (Figure 7).

## Author Contributions

**Xinwen Kuang:** conceptualization (equal), data curation (equal), formal analysis (lead), investigation (lead), writing – original draft (lead). **Zhengyun Liang:** data curation (supporting), investigation (supporting), methodology (supporting), visualization (supporting). **Yijun Xia:** formal analysis (supporting), investigation (equal), methodology (equal). **Mengjie Shan:** conceptualization (supporting), methodology (supporting), writing – review and editing (supporting). **Yan Hao:** data curation (supporting), software (supporting). **Hao Liu:** data curation (supporting), formal analysis (supporting), methodology (supporting). **Zhi Wang:** methodology (equal), project administration (equal), resources (equal). **Cheng Feng:** investigation (supporting), methodology (supporting), resources (supporting). **Guojing Chang:** methodology (supporting), resources (supporting). **Youbin Wang:** conceptualization (lead), funding acquisition (lead), project administration (lead), resources (lead), writing – review and editing (lead). **Qianjun He:** formal analysis (equal), writing – review and editing (equal). **Chao Xia:** formal analysis (equal), writing – review and editing (equal).

## Conflicts of Interest

The authors declare no conflicts of interest.

## Supporting information


**Figure S1.** (A) Representative image of a rabbit treated with VSD. (B) The abscissa is the test sample (*n* = 30), and the ordinate refers to the PC1 value of the corresponding sample in PCA analysis. (C) Density plot illustrating the distribution of metabolite intensity before and after normalisation.

**Table S1.** Primer sequences.

## Data Availability

The data that support the findings of this study are available from the corresponding author upon reasonable request.
