# Supplementary figures and images for: Hydrogen‐Rich Saline Combined With Vacuum Sealing Drainage Promotes Wound Healing by Altering Biotin Metabolism
Source: J Cell Mol Med. 2025 Jan 13;29(1):e70292. doi: 10.1111/jcmm.70292 (PMC11728484; doi:10.1111/jcmm.70292)

A

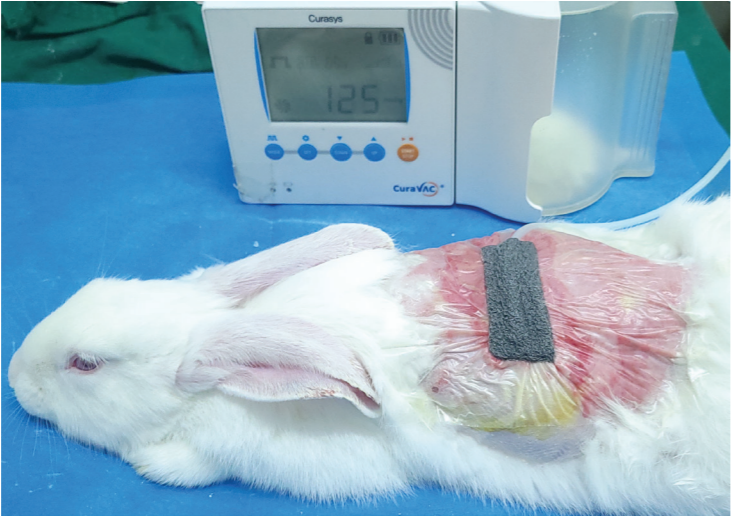

B

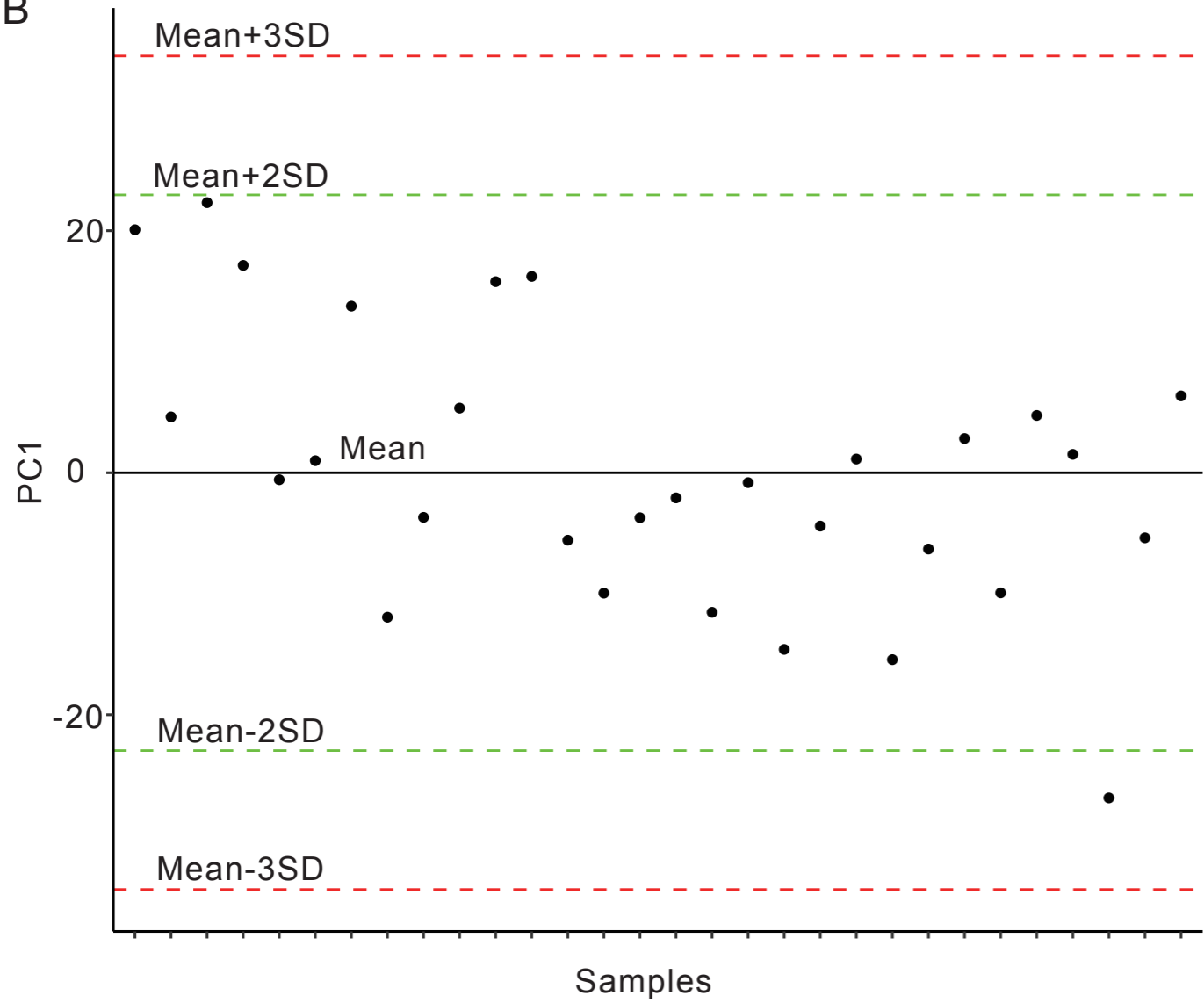

C

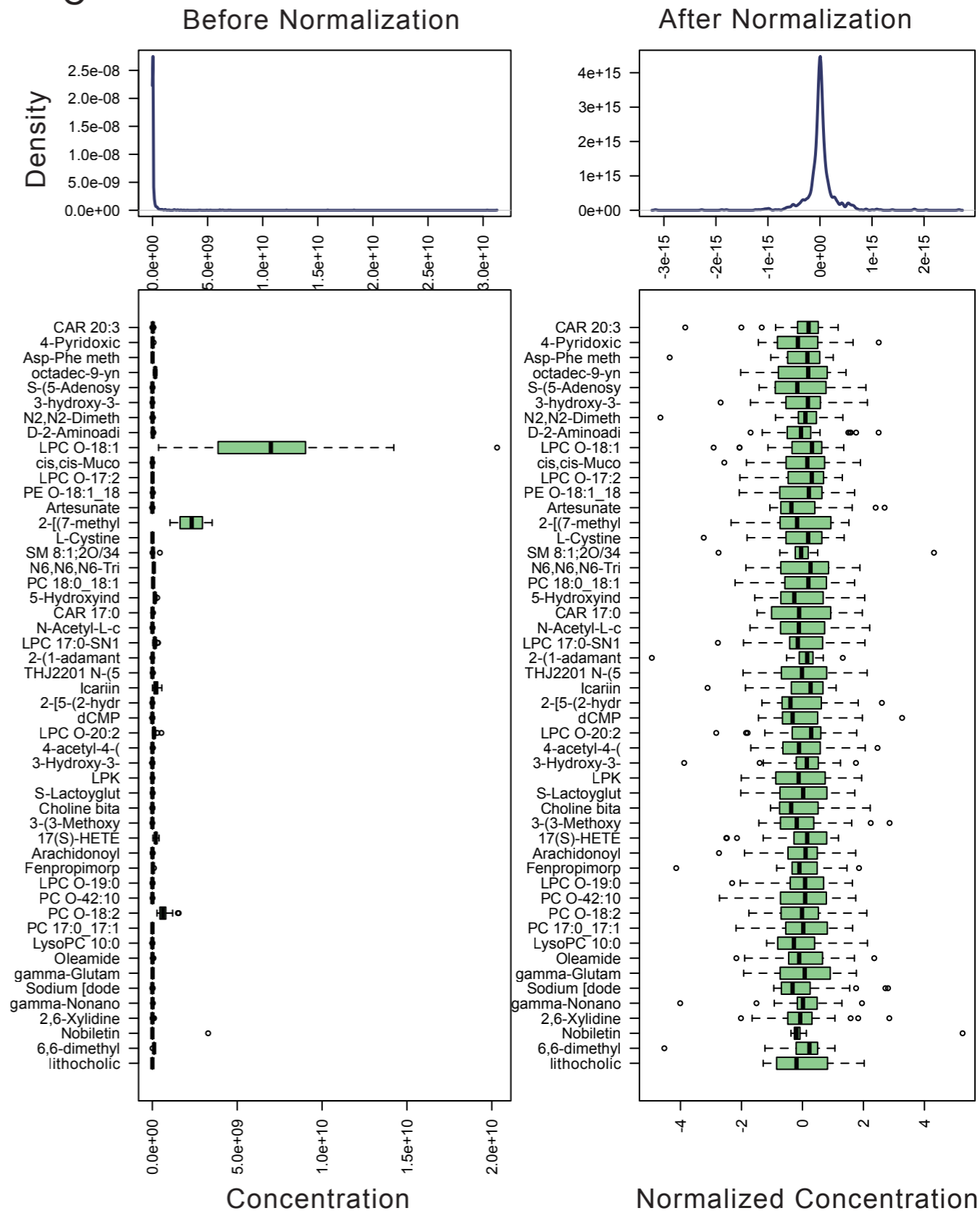

Supplement: Supplementary file 1 — Figure S1. (A) Representative image of a rabbit treated with VSD. (B) The abscissa is the test sample (n = 30), and the ordinate refers to the PC1 value of the corresponding sample in PCA analysis. (C) Density plot illustrating the distribution of metabolite intensity before and after normalisation. [file JCMM-29-e70292-s002.pdf]
