# Supplementary material for: Hydrogen‐Rich Saline Combined With Vacuum Sealing Drainage Promotes Wound Healing by Altering Biotin Metabolism
Source: J Cell Mol Med. 2025 Jan 13;29(1):e70292. doi: 10.1111/jcmm.70292 (PMC11728484; doi:10.1111/jcmm.70292)
Supplement: Supplementary file 2 — Table S1. Primer sequences. [file JCMM-29-e70292-s001.docx]

**Supplementary Table S1.** Primer sequences.

| **Mouse Target Gene** | **Forward Primer** | **Reverse Primer** | **Target Size** |
| --- | --- | --- | --- |
| **β-actin** | CCATGTACGTGGCCATCCAG | TCTTCATGAGGTAGTCGGTCAGGTC | 148nts |
| **IL-1β** | TTGAAGAAGAACCCGTCCTCTG | CTCATACGTGCCAGACAACACC | 128nts |
| **IL-10** | CTTTGGCAGGGTGAAGACTTTC | ACTGGATCATCTCCGACAAGG | 126nts |
| **TNFα** | TGTCTTCACCCCCTCTCGTC | AGGAGGGTGCTCACTAGACC | 135nts |
